# Dielectric anisotropy changes in MBBA liquid crystal doped with barium titanate by a new method

**DOI:** 10.1038/s41598-024-56219-7

**Published:** 2024-03-08

**Authors:** Maryam Beigmohammadi, Mahsa Khadem Sadigh, Jaafar Poursamad

**Affiliations:** Department of Laser and Optics Engineering, University of Bonab, Bonab, Iran

**Keywords:** Materials science, Physics

## Abstract

In response to the burgeoning interest in enhancing the properties of liquid crystal composites, this research systematically explores the intricate interplay between MBBA nematic liquid crystals and ferroelectric barium titanate nanoparticles. The focus is modulating dielectric properties under temperature, frequency, and an applied electric field. Nuanced insights into temperature-dependent behavior, parallel and perpendicular component alterations, and a nonlinear correlation between nanoparticle concentration and dielectric constant are revealed. The study delves into dielectric anisotropy, indicating a reduction with increasing temperature. Structural analyses validate size reduction and crystal phase maintenance of barium titanate nanoparticles (NPs), emphasizing their impact on dielectric characteristics. Frequency-dependent investigations underscore a consistent decline in permittivity with rising frequency across nanoparticle concentrations. Application of an electric field in filling process of liquid crystal cells reveals irregular changes in dielectric constant, holding promise for tailored applications in display technologies. These comprehensive findings offer valuable insights into manipulating dielectric anisotropy properties of MBBA liquid crystal by a simple method for potential advancements in optoelectronic devices and display technologies.

## Introduction

Over the recent decades, scholarly attention has been drawn to investigating composite liquid crystals (LCs) due to their distinct chemical attributes and noteworthy physical and electro-optic properties^1,2^. The introduction of various nanomaterials, including carbon nanotubes, dyes, quantum dots, and polymers, into LCs has enabled the development of novel materials endowed with unique characteristics^3–6^. Substantial research efforts have been devoted to enhancing the physical properties of composite systems within the domain of liquid crystal (LC) composites^7–16^. Notably, the physical attributes of nematic liquid crystals, such as dielectric constant, can be influenced by external factors such as applied field intensity, frequency, and temperature. Consequently, investigations into the characteristics of liquid crystals around the transition temperature valuable insights for designing optical and photonic devices.

The dielectric constant of a focused center in LCs regulates the molecular electro-optical response, anisotropy, and dynamics of the liquid crystal environment^17,18^. Permittivity, denoting the capacity of a material to polarize when exposed to an external electric field, assumes a crucial role. The inherent anisotropy of physical properties in LCs permits altering the direction of the LC molecular axis under the influence of an electric field^19^. Dielectric anisotropy, which significantly impacts the image quality of liquid crystal displays, becomes an essential parameter for display applications.

The compound *N*-(4-methoxybenzylidene)-4-butylaniline (MBBA) stands out as the inaugural liquid crystal manifesting a nematic phase at temperatures between 21 and 44 °C^5^, finding application in the production of liquid crystal displays (LCDs). Due to the nematic phase of MBBA at room temperature, researchers in optoelectronic device technology have been significantly attracted to it^20^. The introduction of diverse nanoparticle types (dielectric, ferroelectric, ferromagnetic, multiferroic, metallic, semiconducting) can profoundly alter the properties of existing LC materials^8–15^, rendering it a subject of sustained interest over the past few decades^21^. Noteworthy observations indicate that even a minimal quantity of nanoparticle doping in nematic LC hosts induces substantial changes in their diverse properties^22,23^.

The burgeoning applications of LC materials in display devices, photonics, optical processing, sensors, lenses, filters, spatial light modulation, and adaptive optics necessitate LC materials with refined properties^24–29^. Despite the intricacies inherent in synthesizing new liquid crystalline materials with unusual properties, low-concentration doping in these materials emerges as a salient alternative for enhancing their physical attributes. The enhanced properties created in the LC material depend on various parameters of the doped material, primarily on its nature, the size and shape of the particles, the number of impurities and the mutual interaction between nanoparticles and LC molecules^30^. It is empirically observed that achieving improved properties in any LC via doping nanoparticles in the pure LC host generally requires minimizing disturbances to the director of the LC host as much as possible. To ensure this, the contaminant concentration in the LC host must be kept low and the impurity particle size must be sufficiently small. The size effects of barium titanate (BaTiO_3_) particles have been systematically documented in various scholarly publications^31–36^.

The impact of an applied electric field on liquid crystals has predominantly been examined through theoretical frameworks and simulations, focusing on investigating its effects on anchoring and director orientation^37–41^. This study aims to investigate the impact of the electric field on liquid crystal cells during the filling process. Specifically, we focus on the MBBA liquid crystal and barium titanate nanoparticles. This research is a new experimental inquiry of its sort in this context. It is crucial to provide an electric field when filling cells for practical purposes, especially in liquid crystal technology. Applying an electric field causes a realignment of liquid crystal molecules during the filling process, a phenomenon widely examined via theoretical frameworks and simulations. Nevertheless, the empirical examination of this phenomenon, particularly within the particular framework of MBBA liquid crystal and barium titanate nanoparticles, still needs to be explored. Gaining a comprehensive understanding of the impact of the electric field during the filling process is crucial for maximizing the efficiency of liquid crystal cells in display applications.

A review of recent literature reveals significant contributions in this domain. For instance, in 2016, Ibragimov et al. investigated the effects of employing 50 nm barium titanate particles in conjunction with nematic liquid crystal H 37, focusing specifically on variations in Fredericks voltage^42^. Subsequently, in 2023, Teekendra Kumar Sahu et al. conducted a study on MBBA liquid crystals under different conditions of field application, ensuring the absence of material contamination^43^. Also, in 2023, Praveen Kumar et al. presented a study examining viscosity dynamics under the influence of an electric field^44^.

In the current study, barium titanate was precisely tailored to achieve the desired size using the ball milling method, representing a novel approach in experimental investigations of the effects of an electric field on the parallel and perpendicular components of liquid crystals. This experimental endeavor adds a practical dimension to the existing theoretical knowledge, providing valuable insights into the dynamic interplay between electric fields and the electrical properties of liquid crystals, particularly in the presence of barium titanate nanoparticles.

## Experimental

### Materials

**Figure 1:**
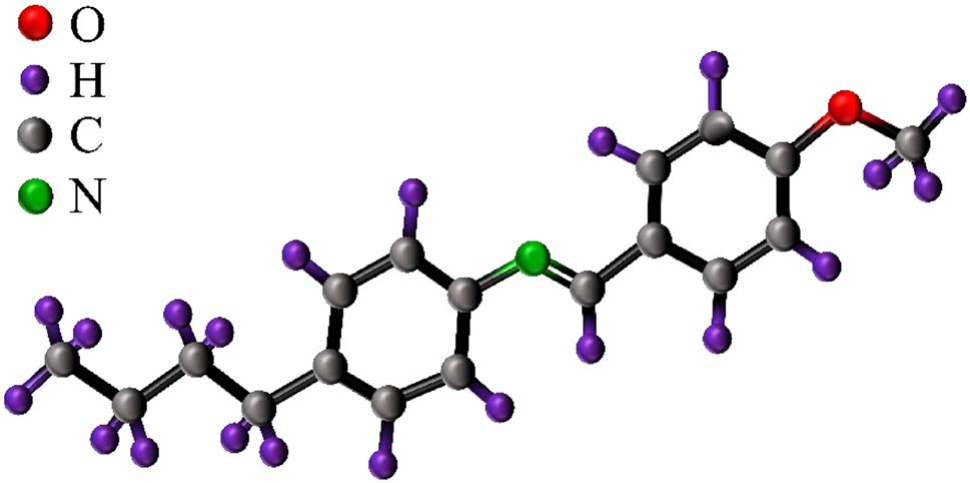
Molecular structure of MBBA liquid crystal.

In the present investigation, the MBBA nematic liquid crystal and barium titanate nanoparticles were procured and employed, sourced from the Sigma Aldrich Company. The molecular configuration of the liquid crystal under examination is depicted in Fig. 1.

### Preparation of LC cell

In the current investigation, planar sandwich-type and homeotropic sample cells were employed. These cells were meticulously fabricated by interposing the samples between two optical glass plates (2 × 1.5 cm^2^), each adorned with indium tin oxide (ITO) layers. The sample cells were aligned by applying a surface layer comprising polyvinyl alcohol with parallel rubbing. Lecithin was employed as a surfactant for the surface layer of liquid crystal (LC) cells, facilitating their regulation in a homeotropic manner. To establish a uniform distance between the electrode surfaces, a Mylar film served as a spacer, maintaining a separation of 17 μm. Ultimately, a sealing material was employed to affix the plates together, ensuring the integrity of the experimental setup.

Moreover, for each of the specified concentrations, we used separate cells in parallel and perpendicular directions. Our investigations show that the results are repeatable for different weight percentages and the obtained results are acceptable.

### Dielectric measurement

An LCR meter (VICTOR 4091C) with temperature control was employed to ascertain the parallel and perpendicular dielectric constants. The experimental setup involved the utilization of a pure nematic liquid crystal doped with varying weight percentages of ${{\text{BaTiO}}}_{3}$ nanoparticles. Dielectric measurements were conducted using a built-in capacitor cell, and capacities were assessed at different frequencies and temperatures. The capacities of the samples were determined in both filled and empty states across diverse temperature ranges.

The parallel (${\varepsilon }_{\parallel }$) and perpendicular (${\varepsilon }_{\perp }$) dielectric constants were calculated using the following expressions:

1 $$ \varepsilon_{\parallel } = \frac{{C_{ \bot } }}{{C_{^\circ } }}\varepsilon_{ \bot } = \frac{{C_{\parallel } }}{{C_{^\circ } }} $$

${C}_{\parallel }$ and ${C}_{\perp }$ represent the aligned liquid crystal capacities in parallel and perpendicular directions to the cell surface, and ${{\text{C}}}_{\circ }$ is the capacity of the corresponding empty cell.

Subsequently, the dielectric anisotropy ($\Delta \varepsilon$) was determined by the equation:

2 $$\Delta \varepsilon ={\varepsilon }_{\parallel }-{\varepsilon }_{\perp }$$

Here, ${\varepsilon }_{\parallel }$ denotes the parallel component (planar), and ${\varepsilon }_{\perp }$ represents the perpendicular component (homeotropic). A positive value of dielectric anisotropy ($\Delta \varepsilon$) indicates that the LC molecules align in the direction of the applied electric field (${\varepsilon }_{\parallel }>{\varepsilon }_{\perp }$). Conversely, a negative $\Delta \varepsilon$ implies that the vertical component surpasses the parallel component in magnitude. In the case of MBBA liquid crystal, a negative dielectric anisotropy is observed.

Our aim in this experimental work is to investigate the dielectric anisotropy of liquid crystal contaminated with barium titanate nanoparticles. For this purpose, considering the Eq. (2), we focused on two parallel and vertical components and did not examine the complex dielectric constant.

### Impedance measurements

In this work, impedance spectra were measured by IVIUMSTAT.h device.

## Results and discussions

### *Characterization of synthesized BaTiO*_*3*_

In producing barium titanate (${{\text{BaTiO}}}_{3}$) ferroelectric nanoparticles, a zirconia ball mill, specifically the Retsch Mixer Mill MM400, was employed to ensure precise and controlled synthesis. The grinding fluid utilized in this process was heptane, which added oleic acid as a surfactant. Incorporating a surfactant was pivotal in preventing the undesirable aggregation of ${{\text{BaTiO}}}_{3}$ nanoparticles, facilitating the achievement of diminutive particle sizes, typically around 10 nm.

The solutions were meticulously prepared following a specific weight ratio of 1:1:20 for ${{\text{BaTiO}}}_{3}$, oleic acid, and heptane, respectively^45^. This well-defined composition was essential to create an optimal environment for synthesizing ${{\text{BaTiO}}}_{3}$ nanoparticles with the desired properties.

**Figure 2:**
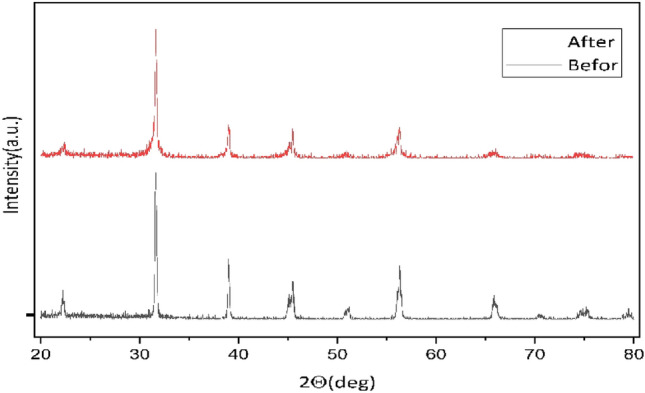
XRD of $${{\text{BaTiO}}}_{3}$$.

To assess the structural characteristics of the ${{\text{BaTiO}}}_{3}$ powders, powder X-ray diffraction (XRD) analyses were conducted using CuKa radiation. The XRD patterns, obtained in continuous mode across the range of 20°–80°, are illustrated in Fig. 2.

The X-ray diffraction (XRD) patterns presented in Fig. 2. provide a comprehensive overview of the structural modifications in the specimens before and after the ball milling procedure. Notably, notwithstanding fluctuations in peak intensity, an unvarying number of peaks has been ascertained between the two specimens, underscoring the preservation of an unadulterated tetragonal structure. The discernment of pronounced peaks at approximately 2θ = 31° holds particular significance, denoting barium titanate’s unaltered crystal phase structure (${{\text{BaTiO}}}_{3}$) in response to the ball milling process. The robustness exhibited in the crystal structure suggests the retention of distinct ferroelectric characteristics associated with the tetragonal phase, thereby accentuating the effectiveness of the synthesis methodology.

**Figure3:**
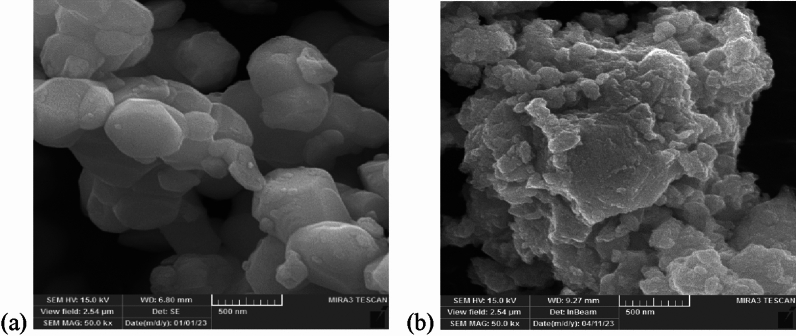
SEM photograph of ${{\text{BaTiO}}}_{3}$ (**a**) before and (**b**) after ball milling.

Concomitant with XRD analyses, insights into morphological alterations induced by the ball milling protocol are gleaned from scanning electron microscopy (SEM) observations, as depicted in Fig. 3. The SEM imagery elucidates a noteworthy reduction in particle dimensions, with the powder manifesting an average particle size of 12 nm after milling. In stark contrast, the powder exhibited a considerably larger mean size of approximately 300 nm before milling. This substantial diminution in particle size is a consequential outcome of the ball milling technique, illustrating its effectiveness in achieving nano-scale dimensions and augmenting the material’s properties. The amalgamation of XRD and SEM analyses perfectly depicts the structural and morphological alterations in the ferroelectric nanoparticles of ${{\text{BaTiO}}}_{3}$ induced by the ball milling process.

The homogeneity and elemental dispersion evaluation within the fabricated nanocomposite was conducted through energy-dispersive X-ray Spectroscopy (EDS) analysis, a technique of considerable utility in delineating the elemental constitution of materials and elucidating spatial element distribution within a given specimen.

**Figure 4:**
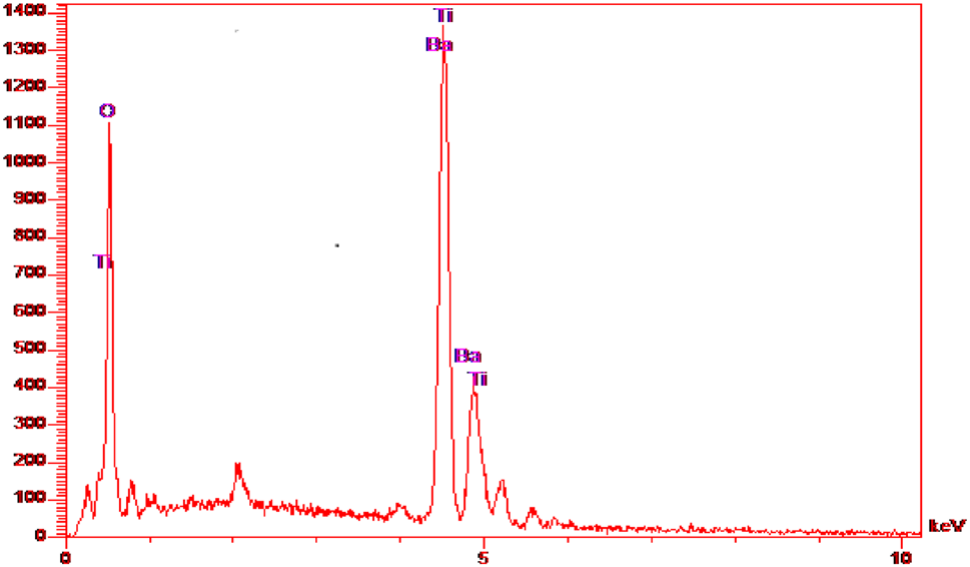
The EDS analysis of ${{\text{BaTiO}}}_{3}.$

As illustrated in Fig. 4, the EDS analysis provides insights into the elemental constituents of barium titanate within the synthesized nanocomposite. This analytical approach facilitates the identification and cartography of individual elements, presenting a visual manifestation of their spatial arrangement throughout the material. The EDS spectrum distinctly depicts the presence and dispersion patterns of crucial elements, such as barium (Ba), titanium (Ti), and oxygen (O), thereby supplying essential data regarding the compositional profile and evenness of the nanocomposite.

Through the application of EDS analysis, researchers acquire a nuanced comprehension of the elemental composition of the nanocomposite, thereby ensuring the attainment of a uniformly distributed array of components through the synthesis process. This information assumes significance in corroborating the synthesis endeavor’s success and evaluating the nanocomposite’s potential efficacy across diverse applications*.*

### Temperature-dependent dielectric permittivity

Precision-engineered cells were employed to introduce mixtures in investigating the dielectric constant of LCs contaminated with nanoparticles across varying concentrations. To control the concentration, we dissolved certain amounts of barium titanate nanoparticle in heptane solvent at a ratio of 1:20 and put it in an ultrasonic bath for 6 h to completely dissolve and have a uniform solution. For different concentrations, we used specific volumes of this solvent according to the weight of the liquid crystal, and we used different weight percentages of nanoparticles. Since heptane solvent has high volatility, perform this step on a heater until the addition of nanoparticles to the liquid crystal does not cause accumulation. For weight percentages greater than 0.5% by weight, the amount of nanoparticle accumulation was seen, so we limited our measurements from low percentages to 0.5% by weight.

**Figure 5:**
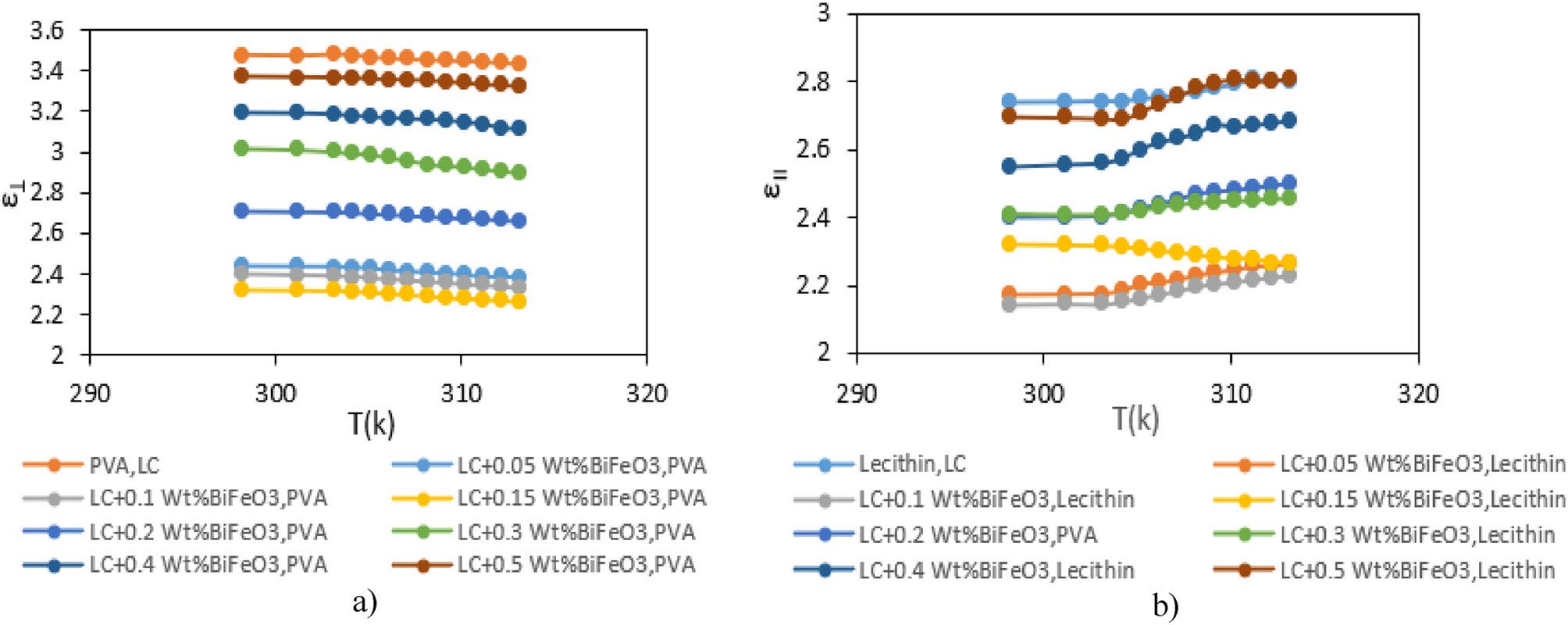
Variation of dielectric constants with temperature for pure MBBA nematic and LC contaminated with variable weight percentages of ${{\text{BaTiO}}}_{3}$, at 20 kHz, (**a**) perpendicular (${\varepsilon }_{\perp }$) and (**b**) parallel (${\varepsilon }_{\parallel }$).

Subsequently, the dielectric constant of these cells was determined for both homeotropic and planar states utilizing Eq. (1). The temperature-dependent fluctuations of the parallel (${\varepsilon }_{\parallel }$) and perpendicular (${\varepsilon }_{\perp }$) components of the dielectric constant are graphically depicted in Fig. 5.

As delineated in Fig. 5, under predominantly parallel alignment, the ${\varepsilon }_{\parallel }$ value manifests an upward trend with increasing temperature, whereas the ${\varepsilon }_{\perp }$ value exhibits a decrement with temperature elevation. Owing to the negative dielectric anisotropy of MBBA, the intrinsic magnitude of ${\varepsilon }_{\perp }$ surpasses that of ${\varepsilon }_{\parallel }$. Additionally, the dielectric constants (${\varepsilon }_{\perp }$ and ${\varepsilon }_{\parallel }$) in instances of nanoparticle contamination are conspicuously inferior to those observed in pure NLC, signifying the nanoparticles’ substantial impact on the liquid crystal’s dielectric characteristics.

Figure 5 portrays the temperature-dependent behavior of dielectric permittivity for pure NLCs and various concentrations at a frequency of 20 kHz. Each concentration is graphically represented by two plots, where the right plot corresponds to the parallel component of dielectric permittivity, and the left one pertains to the component perpendicular to the dielectric permittivity. Notably, the perpendicular component of dielectric permittivity diminishes with escalating temperature, while conversely, the parallel component experiences augmentation.

Introducing a minute quantity of nanoparticles (i.e., 0.05% by weight) reduces the dielectric constant. However, there is a notable escalation in the dielectric constant at higher concentrations. This nonlinear correlation between nanoparticle concentration and dielectric constant underscores the intricate interplay between nanoparticle doping and the dielectric properties of the LC, providing insightful implications for potential applications in advanced optoelectronic devices such as sensors.

### Dielectric anisotropy

The dielectric anisotropy, defined as the disparity between parallel and perpendicular dielectric constants, constitutes a pivotal parameter essential for comprehending the characteristics of liquid crystal materials. In the specific context of introducing barium titanate nanoparticles into MBBA liquid crystal, an exhaustive examination reveals a conspicuous reduction in dielectric anisotropy compared to the pristine nematic liquid crystal.

Dielectric anisotropy in LCs is intricately linked to factors such as molecular polarizability and alterations in the effective dipole moment of LC molecules. Incorporating doped nanoparticles induces a reorientation of the dipole moment relative to the long molecular axis of the LC molecule. The modulation in dielectric anisotropy with varying nanoparticle concentration reflects the cumulative impact of changes in dipole moment and polarizability concerning the parallel (${\varepsilon }_{\parallel }$) and perpendicular (${\varepsilon }_{\perp }$) dielectric constants.

**Figure 6:**
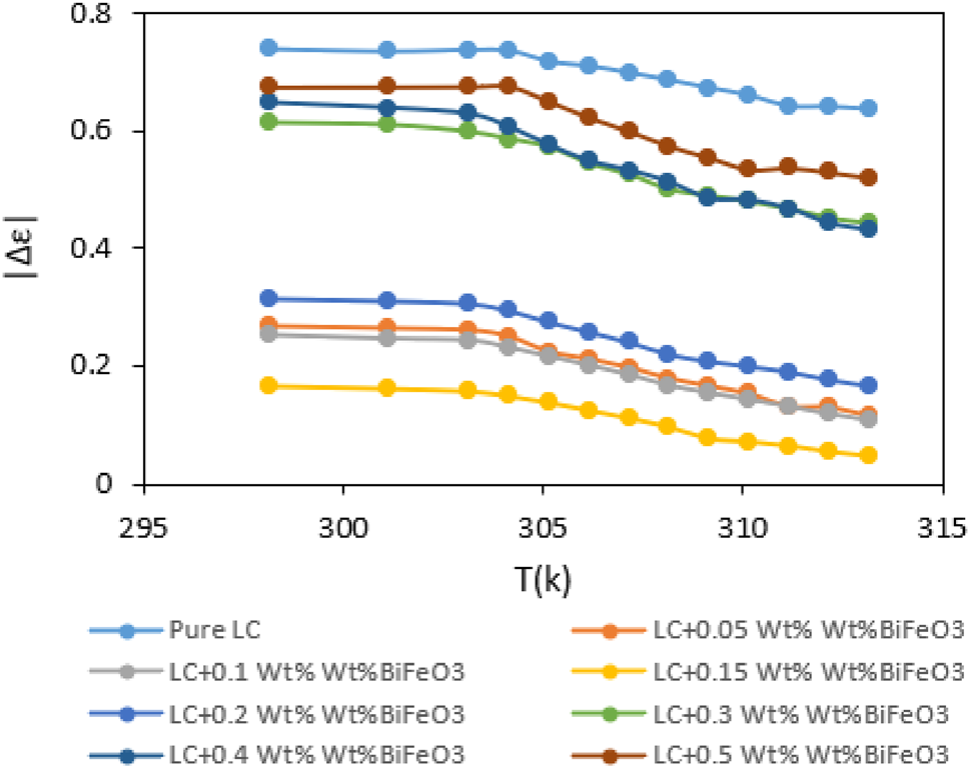
Variation of dielectric anisotropy ($\Delta \varepsilon$) with temperature for LC contaminated with variable weight percentages of ${{\text{BaTiO}}}_{3}$.

Through the judicious addition of an appropriate nanoparticle concentration to a nematic liquid crystal, researchers can systematically regulate the dielectric anisotropy of the LC. Figure 6 visually depicts that the discrepancy between perpendicular and parallel components diminishes with increasing temperature, providing a nuanced insight into the dynamic interplay between temperature and dielectric anisotropy.

Upon scrutiny of Fig. 6, it becomes apparent that the dielectric anisotropy reaches its minimum at a concentration of 0.05% by weight, gradually escalating for higher concentrations. This observation suggests that as nanoparticle concentration increases, the dielectric anisotropy converges towards values akin to the pure samples. In this case, the molecular interactions tend to increase the value of dielectric anisotropy in liquid crystal system. In general, various molecular interactions with different contributions on the orientational order of nematic liquid crystal tend to modify the anisotropy properties of doped liquid crystals. Three molecular interactions can occur:

- 1.LC-LC interactions.
  - 2.LC-NP interactions.
    - 3.NP-NP interactions.

At low concentrations of nanoparticles, the first interactions are dominant. By increasing the concentration of nanoparticles, two other interactions can occur with different contributions in addition to the first interactions. These interactions with different contribution can enhance the orientational order of nematic liquid crystals and finally dielectric constant value. For this reason, dielectric anisotropy for liquid crystal with 0.5 Wt% of BaTiO_3_ is higher than sample with 0.1 Wt% of nanoparticle. Based on Maier and Meier theory, increasing of order parameter led to increasing the dielectric anisotropy.

The graph conveys a modifiable relationship between nanoparticle concentration and dielectric anisotropy, thereby offering a pathway for tailoring the electrical properties of the LC material to suit specific applications.

### Frequency-dependent dielectric permittivity

**Figure 7:**
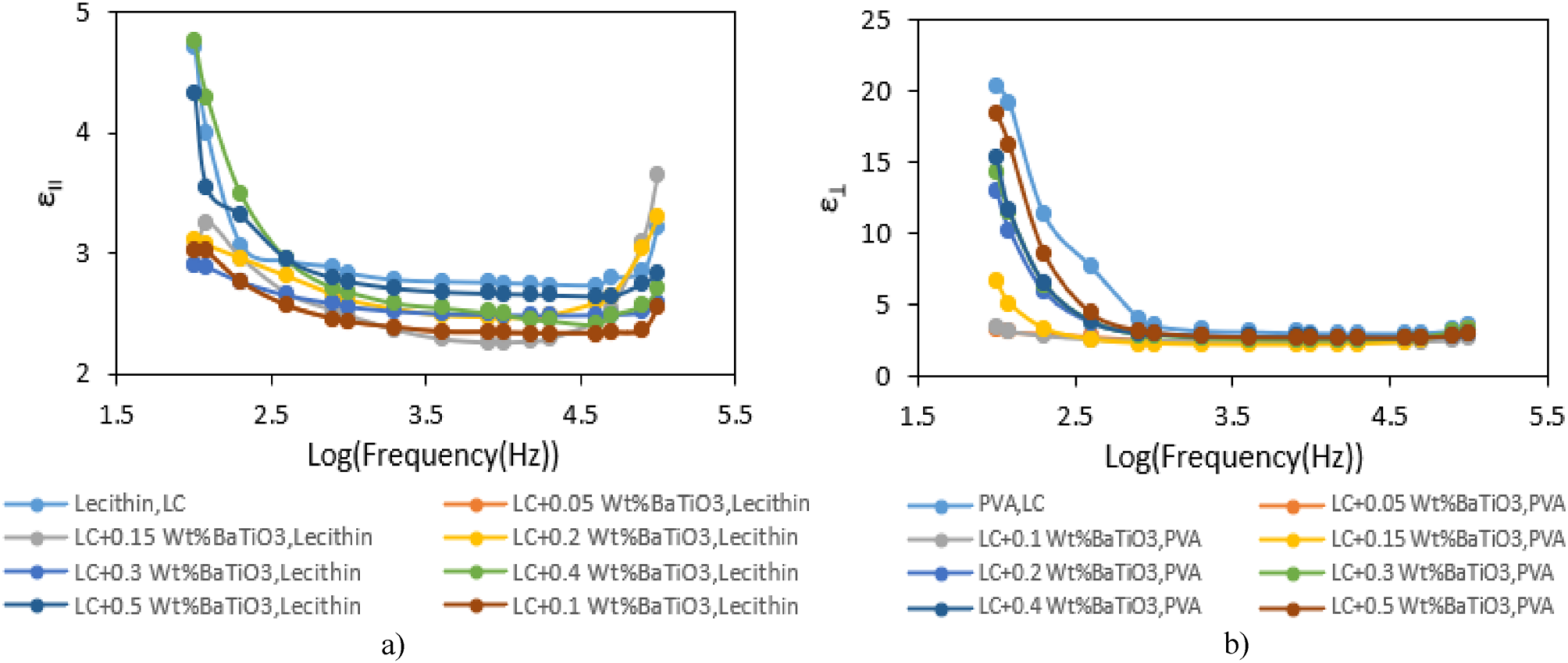
(**a**) ε_װ_, and ε_Ʇ_ changes with frequency in logarithmic scale for pure and doped liquid crystals with different weight percentages of BiFeO_3_^.^

Figure 7 elucidates the frequency-dependent dynamics of the parallel and perpendicular components of dielectric constant within the frequency spectrum ranging from 100 Hz to 0.1 MHz, maintained at a temperature of 35 °C. According to Fig. 7, perpendicular permittivity induces lower frequency dependence related to parallel component. Significantly, the discerned pattern manifests a decline in permittivity with escalating frequency, a trend consistently observed across various concentrations of nanoparticles. This trend aligns coherently with the behavior evident in the pristine state.

Examination of Fig. 7 (a,b) substantiates that introducing nanoparticles induces an influential effect, reducing permittivity. The ε–$f$ plots, delineating the correlation between permittivity (ε) and frequency ($f$), unveil a uniform pattern irrespective of concentration. In the lower-frequency domain, ε remains relatively stable; however, as frequency increases, a conspicuous reduction in permittivity ensues.

This frequency-dependent phenomenon, characteristic in the presence of nanoparticles, signifies that introducing these particulates imparts a discernible impact on the dielectric characteristics of the liquid crystal. The diminution in permittivity with rising frequency implies an intricate interplay between nanoparticle concentration and the response of the LC to the applied frequency. Such discernments are paramount for comprehending and modulating the electrical attributes of the material, holding significant implications for potential applications across diverse technological domains.

### Applied electric field effects during the cell filling with liquid crystal

The distinctive dielectric properties inherent in the molecular structures of LCs constitute a fundamental basis for their widespread utilization in display applications. Among the critical phenomena contributing to their efficacy is the response of LC materials to an applied electric field. The imposition of an electric field during filling process of cells induces a consequential reorientation of LC molecules, thereby serving as a pivotal mechanism in their operational principles.

This induced reorientation is of paramount significance for practical applications as it precipitates the generation of electric dipoles through the dielectric interaction between LC molecules and the applied electric field. The formation of these dipoles is a critical facet of the molecular response exhibited by LC materials when subjected to external stimuli, particularly electric fields. Such a phenomenon assumes a central role in achieving desired optical effects in displays, notably in the precise control of the alignment of LC molecules to modulate light transmission.

**Figure 8:**
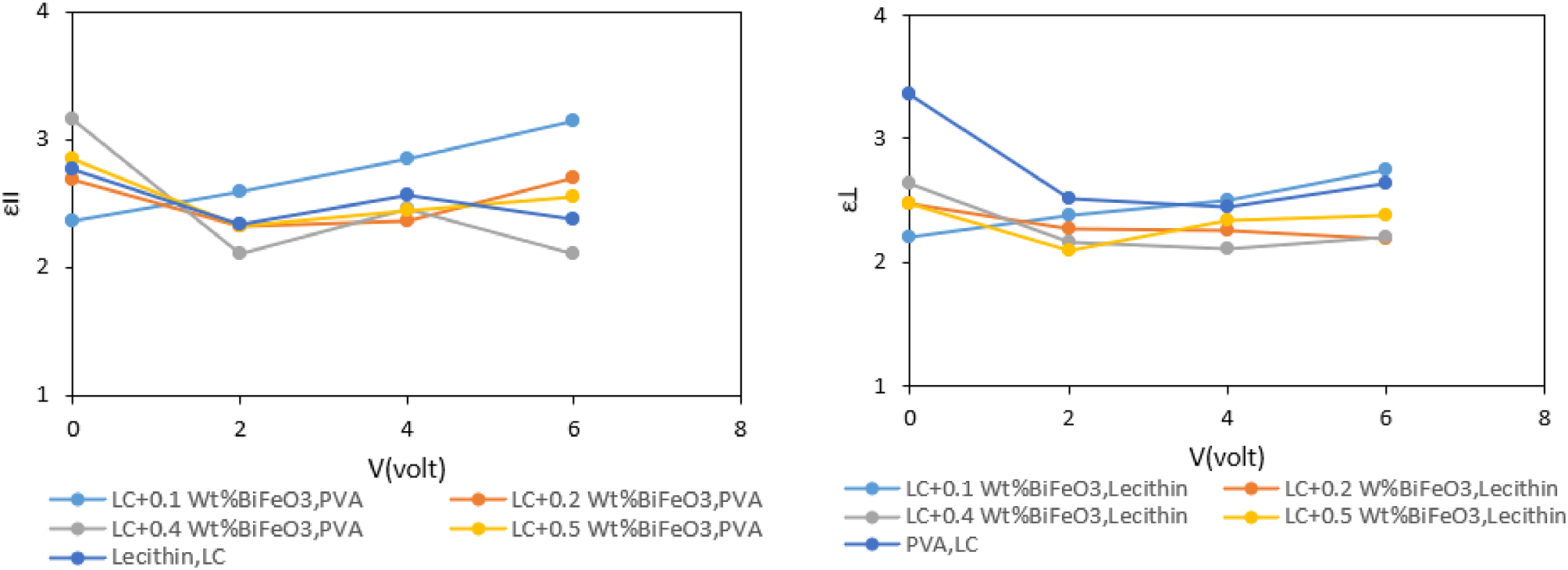
The dependence of the parallel and perpendicular components of dielectric constant to applied voltage for pure MBBA and doped with different weight percentage of BaTiO_3_ nanoparticle.

**Figure 9:**
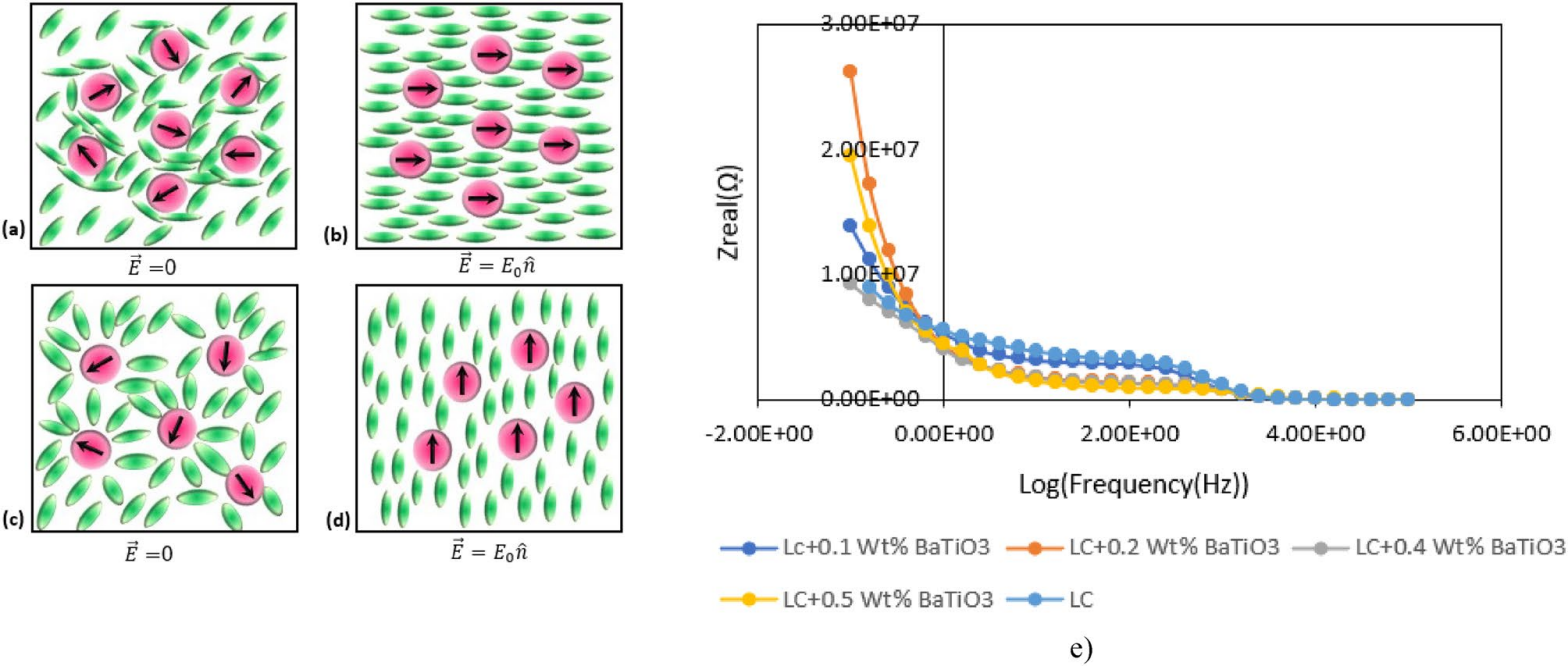
Schematic diagram of the arrangement of LC molecules in a homeotropic cell without applying an external electric field (**a**), with an applied external field (**b**) and in a planar cell without applying an external electric field (**c**), with an external field (**d**) and (**e**) impedance spectra of planar cells with different weight percentage of BaTiO3 nanoparticles.

In order to fully grasp and exploit the capabilities of LC materials in practical applications, a comprehensive examination of their dielectric behavior becomes imperative. The systematic study of dielectric properties facilitates an in-depth assessment of the molecular response of liquid crystals, elucidating the intricate mechanisms that underpin their functionality in a myriad of technological applications, with a particular emphasis on displays. Understanding how LC materials interact with electric fields at the molecular level forms the foundational basis for optimizing their performance and advancing their applications in emergent technologies.Fig. 8. depicts the dependence of dielectric constant changes on the applied voltage for various concentrations. The obtained graphs, derived from applying an electric field within the range of 0–6 V, reveal irregular changes in dielectric components. As can be in Fig. 9, by applying an electric field to parallel and perpendicular cells during cell filling, molecule tend to align along to electric filed (Fig. 9b,d). In this case, the changes in parallel and perpendicular components depend on order parameter and polarizability according to Eq. (3) and Eq. (4) ^46,47^.

3 $$ \varepsilon_{\parallel } = 1 + \frac{Nhf}{{\varepsilon_{0} }}\left\{ {\overline{\alpha } + \frac{2}{3}\Delta \alpha S + \frac{{F\mu^{2} }}{{3k_{b} T}}(1 - (1 - 3\cos^{2} \beta )S} \right\} $$

4 $$ \varepsilon_{ \bot } = 1 + \frac{Nhf}{{\varepsilon_{0} }}\left\{ {\overline{\alpha } - \frac{1}{3}\Delta \alpha S + \frac{{F\mu^{2} }}{{3k_{b} T}}(1 + \frac{1}{2}(1 - 3\cos^{2} \beta )S} \right\} $$

Here, $\overline{\alpha }$ is average molecular polarizability, h is the cavity field factor, μ is the resultant dipole moment of the molecule, N is the molecular number density, F is a parameter dependent on the reaction field factor, S is an order parameter, β is the angle between the molecular axis and the direction of the off axis, and k_b_ is Boltzman constant^46^. According to Meier and Maier’s theory (Eqs. 3 and 4), thermal effects tend to decrease value of dielectric anisotropy.

As can be seen in Fig. 8, the high value for parallel component of dielectric constant is obtained for MBBA liquid crystal with 0.4 Wt% of BaTiO3 without electric field (Fig. 9a,c). By applying electric field, the value of parallel component is changed by increasing the concentration of nanoparticles. At low voltages (2v), the value of parallel component is decreased for MBBA liquid crystal with 0.4 Wt% of BaTiO_3_ in comparison to other liquid crystal cells. Furthermore, this behavior is repeated by increasing applied field. It seems, by increasing the concentration of nanoparticles, dipole–dipole interactions are increased in nanoscale domains that known as pseudo-nematic domains (Fig. 9a–d). In these domains, BaTiO_3_ nanoparticles indicate spontaneous polarization and strong local fields. By moving away from these domains, the local field is decreased. As.

schematically indicated in Fig. 9a–d, these local field lead to orientation of liquid crystal molecules in pseudo-nematic domains. By increasing the concentration of BaTiO_3_, the pseudo- nematic domains and interactions between liquid crystal molecules and nanoparticles increased. Under this condition, the orientation of liquid crystal and order parameter can be modified by change the concentration of nanoparticles. This is in agreement with Meier and Maier’s theory. Hence, the MBBA liquid crystal with high concentration of nanoparticles indicates high value for parallel component of dielectric constant. By applying electric field in cell filling process, the orientation of liquid crystal molecules with electric field can wreak interactions between liquid crystal molecules and nanoparticles. Therefore, the order parameter changes and parallel component of dielectric constant is decreased for MBBA liquid crystal with high concentration of nanoparticles in comparison to other liquid crystal samples. Moreover, the similar discussion can be considered for perpendicular component of dielectric constant.

It can be deduced that the quantity of contaminated nanoparticles and applying electric field in filling process of cells give a new and simple method for controlling the anisotropy of liquid crystal-based systems**.**

For precise investigation, the impedance of parallel cells was measured. According to Fig. 9e, the impedance of cells with 0.4 Wt% of nanoparticles is lower than other cells. Hence, response of liquid crystal samples can be changed by increasing the concentration of liquid crystals.

Our study introduced a technique to control the dielectric anisotropy of liquid crystal. Accuracy in dielectric anisotropy tuning paves the way for new application in liquid crystal technology. In previous works, the field has been applied after filling the cells, and they have mostly focused on conductivity of cells. But in what we did, we apply the field from the initial moment of filling the cells, according to the data obtained from this method, the value of anisotropy can be controlled. This is important because this anisotropy is essential for controlling the transmission of light in devices such as displays^48^.

Moreover, a significant enhancement highlighted in our research is the meticulous control over the size of barium titanate nanoparticles via the ball milling technique and its demonstrated impact on the dielectric properties of liquid crystals. This advance overcomes the limitations of earlier works, where the management of nanoparticle size and its effects on material properties were not as rigorously detailed. Our approach emphasizes the paramount importance of nanoparticle size in optimizing liquid crystal composite properties, setting a new standard for material science research^49,50^.

## Conclusions

In this study, we systematically explored the impact of barium titanate at varying concentrations on the dielectric properties of MBBA nematic LC. The analysis of the permittivity and dielectric anisotropy variation diagram revealed a temperature-dependent trend where the permittivity decreased with rising temperature, culminating in a reduction in the difference between parallel and perpendicular permittivity-commonly referred to as dielectric anisotropy. The investigation into the frequency dependence of permeability demonstrated an initial decrease followed by an increase. Notably, our findings indicated an enhancement in dielectric anisotropy with increasing nanoparticle concentration up to 0.5 by weight, although the values remained lower than those observed in the pure state.

Based on our results, the MBBA liquid crystal with 0.5% by weight of BaTiO_3_ exhibited significantly elevated dielectric anisotropy compared to the pure nematic liquid crystal. Furthermore, the dielectric anisotropy exhibited variations in liquid crystals with higher weight percentages of nanoparticles at different temperatures. Additionally, we investigated the alterations in the electric field ranging from 0 to 6 V. In this case, the value of dielectric anisotropy can be controlled by applying electric field in filling process.

## Data Availability

All data generated or analyzed during this study are included in this published article.
